# Predicting early postoperative PONV using multiple machine-learning- and deep-learning-algorithms

**DOI:** 10.1186/s12874-023-01955-z

**Published:** 2023-05-31

**Authors:** Cheng-Mao Zhou, Ying Wang, Qiong Xue, Jian-Jun Yang, Yu Zhu

**Affiliations:** 1 Department of Anaesthesiology, Central People’s Hospital of Zhanjiang, Zhanjiang, Guangdong China; 2 Anesthesia and Big Data Research Group, Central People’s Hospital of Zhanjiang, Zhanjiang, Guangdong China; 3 Department of Anesthesiology, Pain and Perioperative Medicine, First Affiliated Hospital of Zhengzhou University, Zhengzhou, Henan China

**Keywords:** PONV, Machine learning, Deep learning, SVC, AUC

## Abstract

**Objective:**

PONV reduces patient satisfaction and increases hospital costs as patients remain in the hospital for longer durations. In this study, we build a preliminary artificial intelligence algorithm model to predict early PONV in patients.

**Methods:**

We use R for statistical analysis and Python for the machine learning prediction model.

**Results:**

Average characteristic engineering results showed that haloperidol, sex, age, history of smoking, and history of PONV were the first 5 contributing factors in the occurrence of early PONV. Test group results for artificial intelligence prediction of early PONV: in terms of accuracy, the four best algorithms were CNNRNN (0.872), Decision Tree (0.868), SVC (0.866) and adab (0.865); in terms of precision, the three best algorithms were CNNRNN (1.000), adab (0.400) and adab (0.868); in terms of AUC, the top three algorithms were Logistic Regression (0.732), SVC (0.731) and adab (0.722). Finally, we built a website to predict early PONV online using the Streamlit app on the following website: (https://zhouchengmao-streamlit-app-lsvc-ad-st-app-lsvc-adab-ponv-m9ynsb.streamlit.app/).

**Conclusion:**

Artificial intelligence algorithms can predict early PONV, whereas logistic regression, SVC and adab were the top three artificial intelligence algorithms in overall performance. Haloperidol, sex, age, smoking history, and PONV history were the first 5 contributing factors associated with early PONV.

**Supplementary Information:**

The online version contains supplementary material available at 10.1186/s12874-023-01955-z.

## Highlights

1 This is the first study to construct a predictive model for early PONV in patients using a variety of machine learning and deep learning techniques.

2 The study showed that artificial intelligence algorithms can predict early PONV, but logistic regression, SVC and adab were the three artificial intelligence algorithms with the best overall performance.

3 Finally, we built a website to predict early PONV online using the Streamlit app on the website: (https://zhouchengmao-streamlit-app-lsvc-ad-st-app-lsvc-adab-ponv-m9ynsb.streamlit.app/).

**Supplementary Information:**

The online version contains supplementary material available at 10.1186/s12874-023-01955-z.

## Introduction

Postoperative nausea and vomiting (PONV), a protective reflex where gastric contents are rapidly ejected from the oral cavity, is caused by septal muscle contraction after the stimulation of nerve and septal muscle. PONV is a common postoperative complication, usually occurring within 24 to 48 h after surgery, though it may also occur after 5 days. PONV incidence ranges from 20 to 30%, with that of some high-risk patients being as high as 70 to 80% [1]. PONV can reduce patients’ satisfaction with surgical anesthesia, prolong their stay in the recovery room after anesthesia and hospitalization, and thus increase hospitalization costs. It can also lead to serious complications, such as postoperative wound dehiscence, subcutaneous emphysema, pneumothorax, aspiration pneumonia, and disturbance of the internal environment [2].

Though preventive and combined medication have been clinically applied to treat PONV, its occurrence remains common. A study revealed that despite having undertaken continuing medical education, anesthesiologists have not significantly improved their skills in PONV prevention or treatment, nor have they been able to adequately prevent and treat high-risk PONVs [3]. Thus, it is evident that flawed PONV prediction models and complex clinical anti-vomiting preventive tactics have become the paramount obstacle in this area [4].

Artificial intelligence has drawn increasing attention within the medical field as clinical practice and personalized treatment continues to improve [5–7]. In this study, machine learning and deep learning were used to evaluate early PONV, and to establish a model to predict early PONV. This is important for identifying high-risk patients, improving the understanding of early PONV, and facilitating clinical treatment.

## Methods

### Study design and ethical approval of the study

This is a retrospective analytical study. It was approved by the Ethics Committee at the First Affiliated Hospital of Zhengzhou University (No. 2021-KY-645). All methods were conducted in accordance with relevant guidelines and regulations, and the study was conducted in accordance with the Declaration of Helsinki. As this was a secondary retrospective study using a database, the ethics committee exempted the requirement for informed consent. Data was obtained from the BioStudies public database, and patient information was anonymized and de-identified prior to access and analysis.

### Patients

All patients who underwent general anesthesia and surgery at First Affiliated Hospital of Zhengzhou University between July 1, 2008 and June 19, 2012 were included in this study. All patients were at least 18 years of age and had undergone gynecological, urological, or general surgical procedures. Patients were identified as early PONV if they had received ondansetron as a rescue therapy antiemetic after recovery room surgery [8]. In this case, “early” refers to the time period from extubation to PACU discharge [9].

### Machine learning method and model construction

We used Python to develop and validate diagnostic models using machine learning and deep learning technologies. Ten machine learning and deep learning artificial intelligence algorithms—logistic regression, decision tree, linear support vector (SVC), Gaussian naive Bayes (gnb), K-nearest neighbors (knn), AdaBoost (adab), artificial neural networks (ANN), recurrent neural networks (RNN), long short-term memory (LSTM) and convolutional neural network + recurrent neural networks (CNNRNN)—were included.

Logistic regression is a machine learning algorithm for classification problems. The aim of logistic regression is to predict new samples’ categories by modeling the relationship between input features and output categories. The training process for logistic regression algorithms usually entails maximum likelihood estimation to determine model parameters. The goal of maximum likelihood estimation is for the predicted probability of the model to be as close as possible to the actual class label.

Decision tree is a machine learning algorithm for classification and regression problems. The aim of a decision tree is to create a tree structure by dividing the input features layer-by-layer. Each non-leaf node represents a feature, and each leaf node represents a category or value. The process of building a decision tree usually entails a greedy algorithm, that is—each time, a feature that maximizes the information gain after partitioning is selected as a node.

Linear support vector machine (SVM) is a machine learning algorithm for classification and regression problems. The aim of linear support vector machines is to select the nearest hyperplane as the decision boundary among all the hyperplanes that can separate different classes of data. This distance is often called an “interval”, while the nearest hyperplane is called a “maximum interval hyperplane”.

K-nearest neighbors (KNN) is a common machine learning algorithm for classification and regression problems. The aim of the KNN algorithm is to find the *K* nearest known samples for a given unknown sample, and then predict the category of the unknown samples based on the category of the *K* samples. This distance is usually calculated using measures such as Euclidean distance or Manhattan distance.

Artificial neural network (ANN) is a computational model that simulates biological neural networks and is used to solve complex problems such as classification, regression and clustering. It consists of multiple neurons, each of which receives multiple input signals and converts them into output signals through an activation function to process and transmit information. The basic structure of an artificial neural network includes an input layer, hidden layer and output layer. The input layer receives external input, the hidden layer processes the input signal, and the output layer produces the final output. Hidden layers can have multiple layers, and this is called a deep neural network (DNN).

A recurrent neural network (RNN) is an artificial neural network with a memory function. The basic structure of RNN includes an input layer, a hidden layer and an output layer. The neurons in the hidden layer combine the current input with the output of the previous time step through an activation function to produce a new output. These outputs are called hidden states, and can be used as inputs for the next time step. Therefore, the hidden state of RNN can be seen as the network’s memory of the sequence data.

Long short-term memory (LSTM) is a recurrent neural network (RNN) structure which is improved to solve the problem of gradient disappearance and gradient explosion in RNN, so as to improve sequence modeling. Neurons in LSTM consist of three gates (input, output, and amnesia) and a memory unit. These gates control the flow and storage of information through non-linear functions, such as sigmoid functions, to model and remember data.

The main feature of a convolutional neural network (CNN) is the combination of convolution and pooling layers, which can facilitate the extraction of features from input data and their processing in classification or regression tasks. The convolution layer is the core component of a convolution neural network. It extracts features from input data by convolution operation. The pooling layer is used to reduce the feature map’s dimension and computational effort. Convolutional neural networks usually consist of multiple convolution layers, pooling layers and fully connected layers.

Gaussian naive Bayes (GNB) is a machine learning classification technique based on probability methods and the Gaussian distribution. Naive Bayesian assumes that each parameter (also known as a feature or predictive variable) has an independent ability to predict the output variable. The prediction combination of all the parameters is the final prediction, which returns the probability that the dependent variable is classified into each group, and the final classification is assigned to the groups with higher probability (classes).

The steps to establish a prediction model for postoperative nausea and vomiting via an artificial intelligence algorithm are:

1. Data collection: Collect information about patients and build data sets. At the same time, randomly divide the data set into a training set and test set at a ratio of 7 to 3.
2. Data cleaning and pre-processing: Clean and pre-process the collected data, including missing values processing and data normalization.
3. Feature extraction: Extract features from the data and sort the weights.
4. Model training: Use ten artificial intelligence algorithms to train the prediction models.
5. Model evaluation and optimization: To improve the model’s accuracy and robustness, use five-fold cross-validation to evaluate and optimize the model. Adjust the parameters by hand and grid. Evaluate the model’s validity using accuracy, precision, recall, AUC, and F1 values. Verify the model’s validity in the test group.

### Statistical analysis

R language statistical software was applied for data analysis. The measurement data was expressed as mean ± standard deviation if it was normally distributed, and as median and quartiles if it was non-normally distributed. The count data was expressed as percentages (%), and a chi-square test was used for comparisons between groups. The logistic stepwise review model was used for multi-factor analysis, and the difference was considered statistically significant at *P* < 0.05.

## Results

### General information

A total of 2,617 patients were ultimately included in this study. There was no statistical difference in age between the PONV and the non-PONV group. However, there was a statistically significant difference in sex between the two groups (< 0.001) (Table 1).

**Table 1:** Basic information of patients

PONV	No	Yes	*P*-value
Number	2279	338	
Age [years]	61.0 ± 13.2	59.9 ± 14.4	0.265
Sex			< 0.001
Male	1269 (55.7%)	109 (32.2%)	
Female	1010 (44.3%)	229 (67.8%)	
Smoking			0.216
No	1916 (84.1%)	293 (86.7%)	
Yes	363 (15.9%)	45 (13.3%)	
Haloperidol			0.617
No	1167 (51.2%)	178 (52.7%)	
Yes	1112 (48.8%)	160 (47.3%)	
History.PONV			0.004
No	2231 (97.9%)	322 (95.3%)	
Yes	48 (2.1%)	16 (4.7%)	
Combined.Anesthesia			< 0.001
No	1284 (56.3%)	117 (34.6%)	
Yes	995 (43.7%)	221 (65.4%)	
Sufentanil.Epidural			< 0.001
0 μg	1350 (59.4%)	125 (37.0%)	
10 μg	909 (40.0%)	206 (60.9%)	
20 μg	12 (0.5%)	7 (2.1%)	
Piritramide [mg]	2.9 ± 5.9	4.4 ± 7.9	< 0.001
Anesthesia.Duration [min]	177.2 ± 85.9	229.6 ± 96.2	< 0.001
Sufentanil.Infusion [μg/h]	0.4 ± 4.6	0.4 ± 4.0	0.715
Sufentanil.Bolus [μg]	33.6 ± 26.5	34.2 ± 27.3	0.681
Sufentanil.TCI [μg]	1.9 ± 12.6	2.8 ± 19.5	0.548
Remifentanil.Infusion [μg/h]	166.7 ± 358.2	170.8 ± 368.7	0.952
Remifentanil.Bolus [μg]	0.5 ± 7.6	0.4 ± 6.5	0.572
Remifentanil.TCI [μg]	251.5 ± 685.7	350.0 ± 832.1	0.230

### Correlation and characteristic engineering analysis of early PONV

**Fig. 1:**
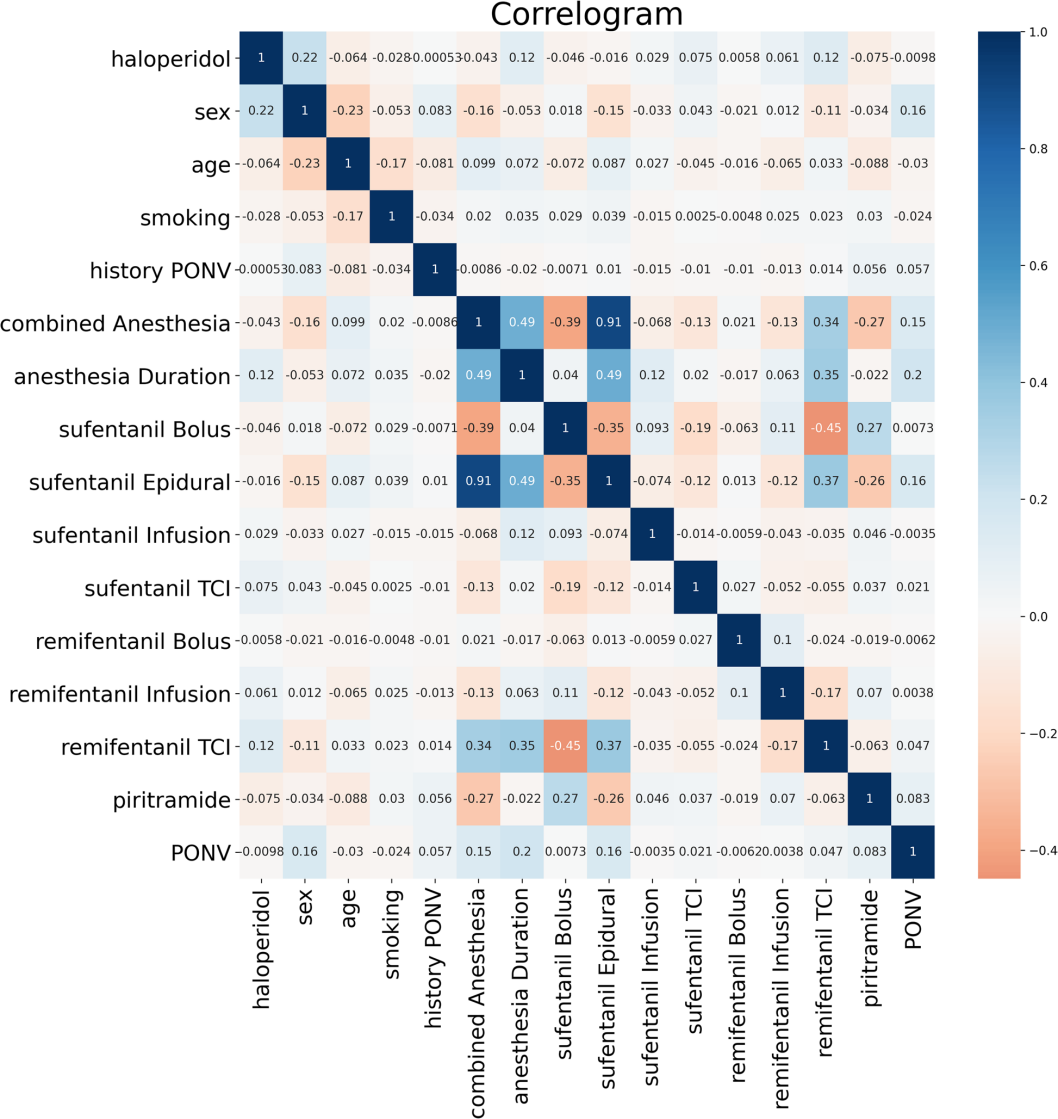
Correlation between clinical characteristic data

We analyzed the correlation and made a heat map. In the heat map, the more relevant, the darker the color, and the specific correlation value is also shown in the map. Correlation analysis showed that the anesthesia duration was positively correlated with early PONV, and age was negatively correlated with early PONV (Fig. 1).

**Fig. 2:**
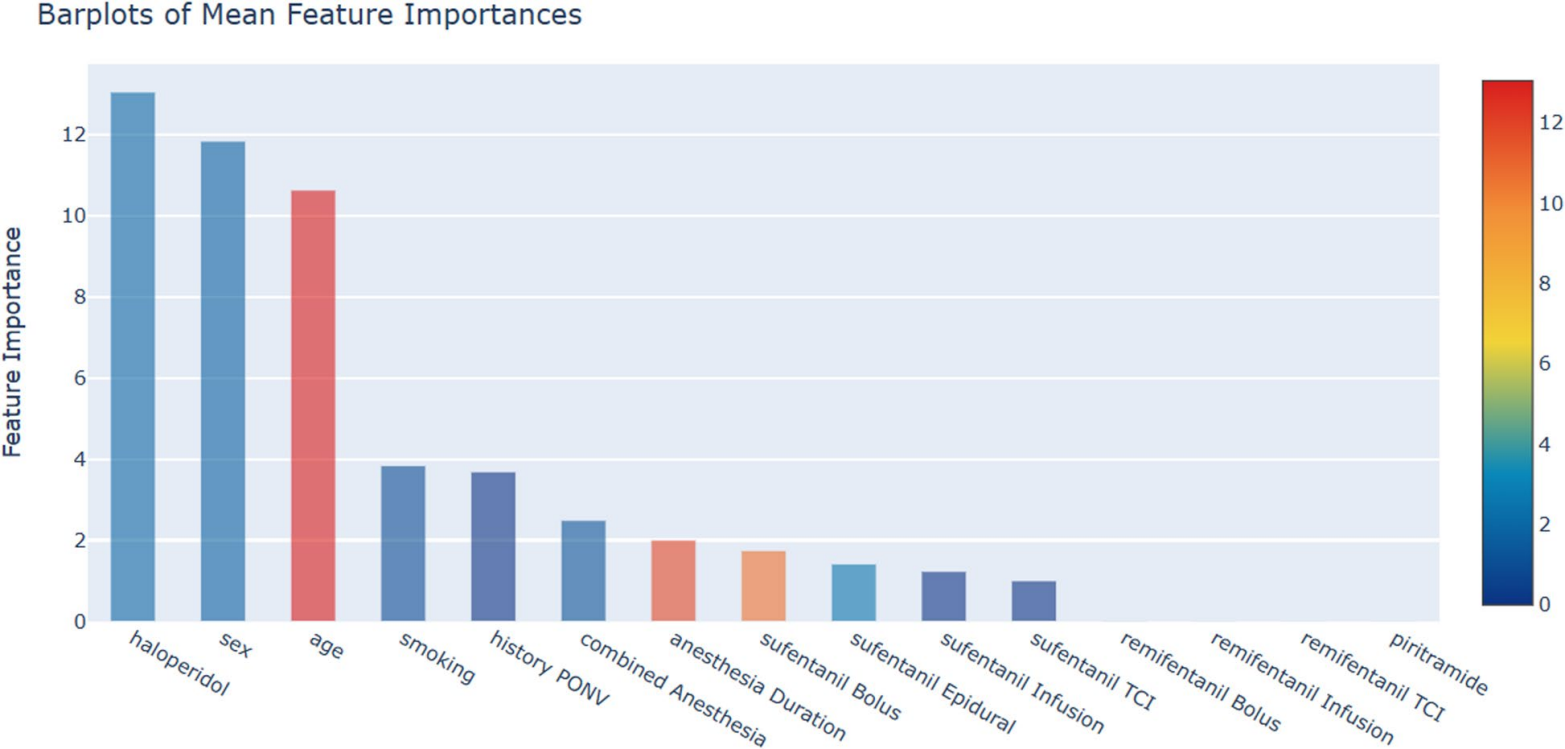
Ranking results of feature weights of average algorithm

In the feature drawing, the variables are ordered from left to right, from high to low, according to their weight in the PONV effect. Average characteristic engineering results showed that haloperidol, sex, age, history of smoking, and history of PONV were the first 5 contributing factors in the occurrence of early PONV in the patients (Fig. 2).

The relevant parameters for some AI algorithms are as follows: LogisticRegression - (penalty = ‘l2’, tol = 0.0000001, C = 10, fit_intercept = True, intercept_scaling = 1, max_iter = 100, multi_class = ‘ovr’, verbose = 0,warm_start = False, n_jobs = 1); Decision TreeClassifier – (splitter = ‘best’, max_depth = 3, min_samples_split = 60, min_samples_leaf = 20, min_weight_fraction_leaf = 0.001, random_state = 1); LinearSVC – (penalty = ‘l2’, loss = ‘squared_hinge’, dual = True, tol = 0.0001, C = 1.0, multi_class = ‘ovr’, fit_intercept = True,intercept_scaling = 1, class_weight = None, verbose = 0, random_state = 1, max_iter = 1,000); K-Neighbors - (n_neighbors = 5, weights = ‘uniform’, algorithm = ‘auto’, leaf_size = 30, p = 2, metric = ‘minkowski’, metric_params = None, n_jobs = 1). The parameters of the remaining algorithms are shown in attachment Supplementary document 1.

### Training group results for predicting early PONV

**Fig. 3:**
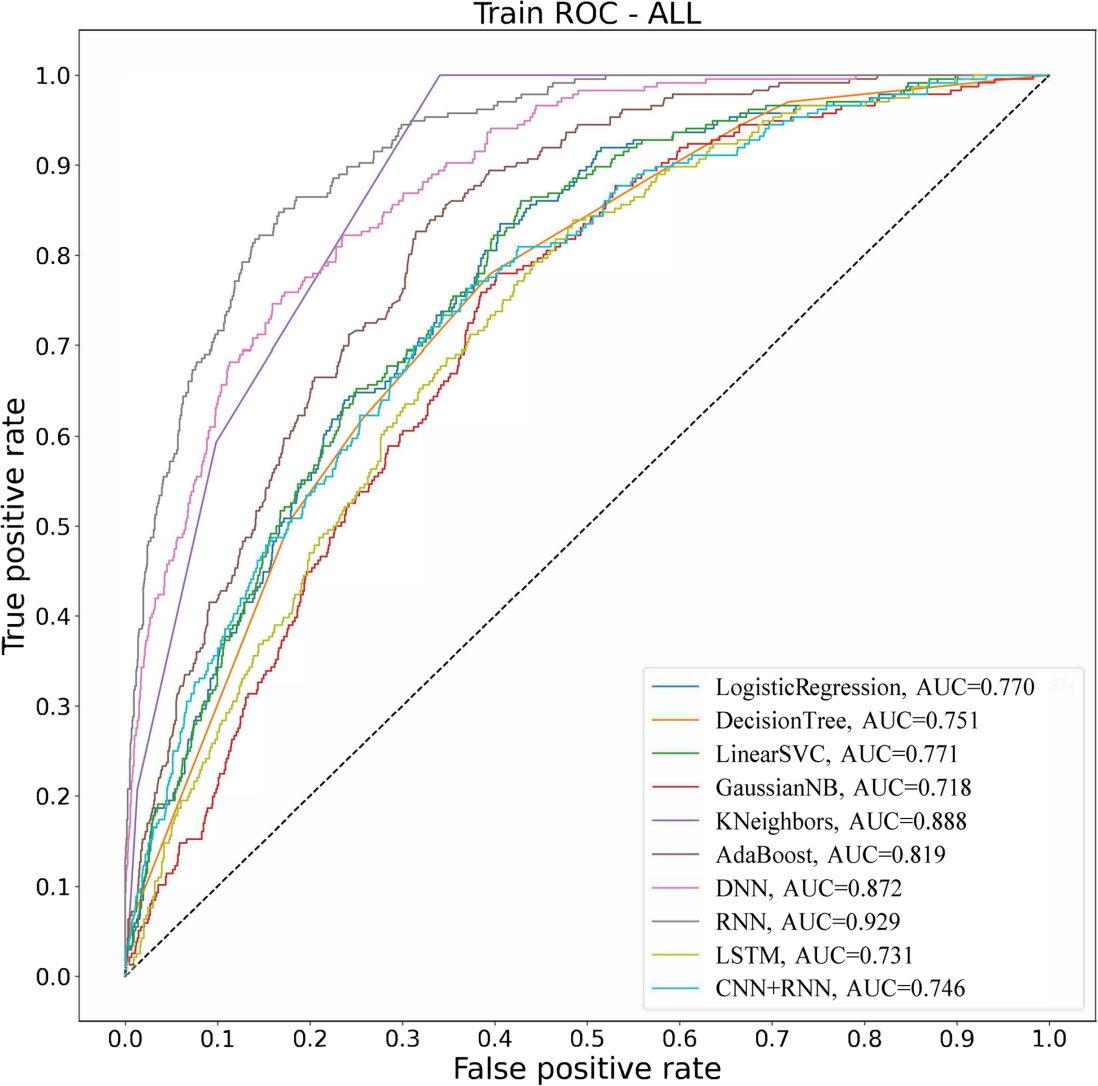
Artificial intelligence algorithm results of training group

In terms of accuracy, the three best algorithms were RNN (0.907), knn (0.886), and ANN (0.886); in terms of precision, the two best algorithms were LSTM (1.000) and CNNRNN (0.800); in terms of recall, the two best algorithms were RNN (0.428) and knn (0.208); in terms of F1 score, the two best algorithms were RNN (0.543) and knn (0.320); in terms of AUC, the two best algorithms were RNN (0.929) and knn (0.888) (Table 2 and Fig. 3).

**Table 2:** Results of artificial intelligence algorithm forecasting early PONV in training group

Train Model name	Accuracy	Precision	Recall	F1 score	AUC
Logistic Regression	0.870	0.464	0.055	0.098	0.770
Decision Tree	0.873	0.583	0.059	0.108	0.751
SVC	0.872	0.583	0.030	0.056	0.771
gnb	0.819	0.222	0.161	0.187	0.718
knn	0.886	0.700	0.208	0.320	0.888
adab	0.871	0.487	0.081	0.138	0.819
DNN	0.886	0.765	0.165	0.272	0.872
RNN	0.907	0.743	0.428	0.543	0.929
LSTM	0.872	1.000	0.004	0.008	0.731
CNNRNN	0.873	0.800	0.017	0.033	0.746

*SVC* Logistic Regression, Decision Tree, Linear Support Vector, *gnb* Gaussian naive Bayes, *knn* K-nearst neighbors, *adab* AdaBoost, *DNN* Artificial Neural Networks, *RNN* Recurrent Neural Networks, *LSTM* Long Short - Term Memory, *CNNRNN* Convolutional Neural Network + Recurrent Neural Networks

### Test group results for predicting early PONV

**Fig. 4:**
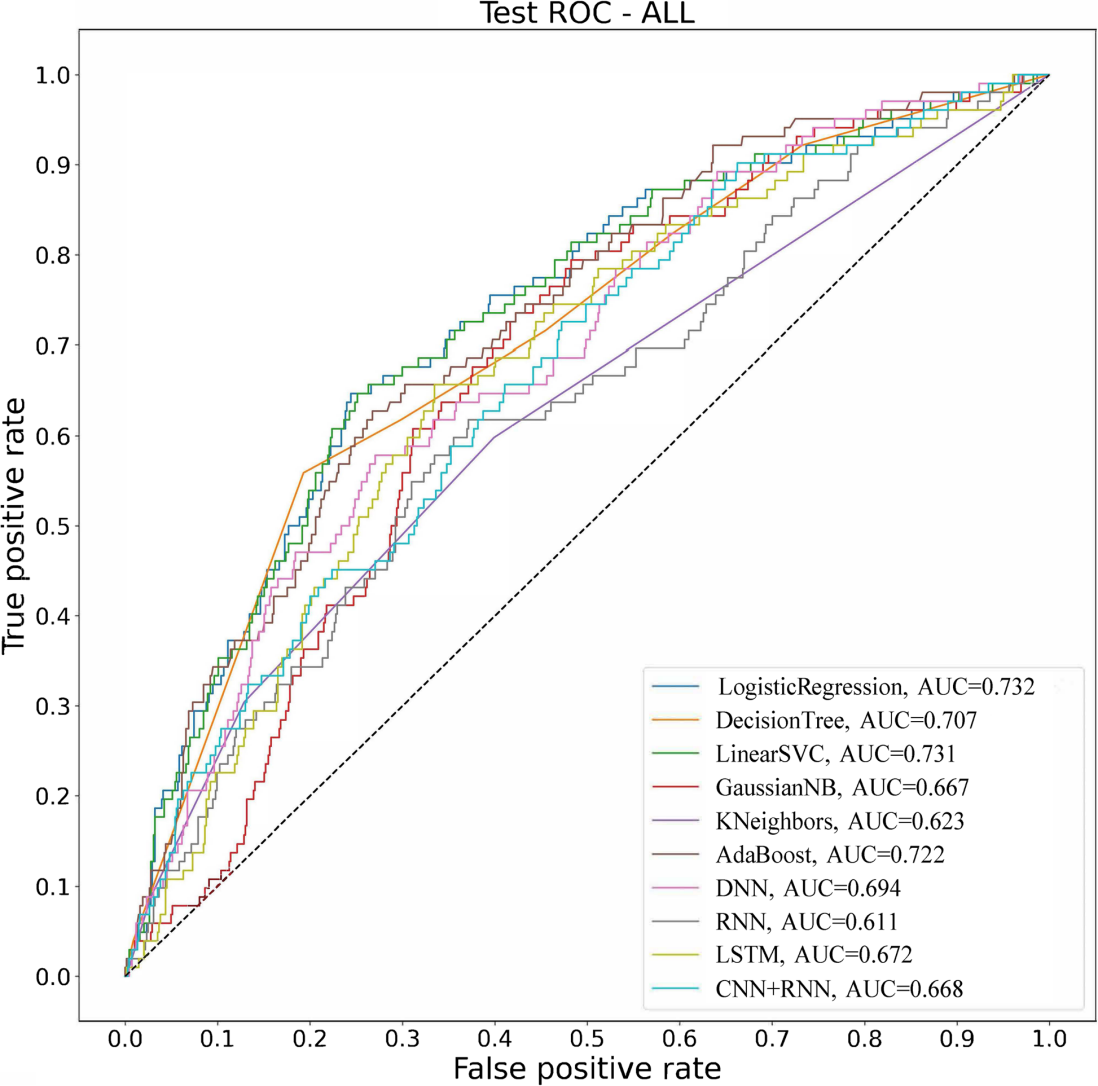
Artificial intelligence algorithm results of testing group

In terms of accuracy, the four best algorithms were CNNRNN (0.872), decision tree (0.868), SVC (0.866) and adab (0.865); in terms of precision, the three best algorithms were CNNRNN (1.000), adab (0.400) and adab (0.868); in terms of recall, the two best algorithms were RNN (0.147) and gnb (0.137); in terms of F1 score, the two best algorithms were RNN (0.170) and gnb (0.144); in terms of AUC, the three best algorithms were logistic regression (0.732), SVC (0.731) and adab (0.722) (Table 3 and Fig. 4).

**Table 3:** Results of artificial intelligence algorithm forecasting early PONV in test group

Test Model name	Accuracy	Precision	Recall	F1 score	AUC
Logistic Regression	0.864	0.308	0.039	0.070	0.732
Decision Tree	0.868	0.400	0.039	0.071	0.707
SVC	0.866	0.333	0.029	0.054	0.731
gnb	0.788	0.151	0.137	0.144	0.667
knn	0.858	0.321	0.088	0.138	0.623
adab	0.865	0.400	0.078	0.131	0.722
DNN	0.861	0.294	0.049	0.084	0.694
RNN	0.814	0.203	0.147	0.170	0.611
CNNRNN	0.872	1.000	0.010	0.019	0.668

*SVC* Logistic Regression, Decision Tree, Linear Support Vector, *gnb* Gaussian naive Bayes, *knn* K-nearst neighbors, *adab* AdaBoost, *DNN* Artificial Neural Networks, *RNN* Recurrent Neural Networks, *LSTM* Long Short - Term Memory, *CNNRNN* Convolutional Neural Network + Recurrent Neural Networks

In terms of integrated performance, logistic regression, SVC, and ADAB performed best among these algorithms. Moreover, in both the training group and the test group, the AUC values for the logistic regression were greater than 0.730, the accuracy rates were greater than 0.860, etc., and the accuracy exceeded 0.300; the AUC values of the SVC were all greater than 0.730, the accuracy rates were all greater than 0.860 etc., and all accuracies were greater than 0.300; the AUC values of ADAB were all greater than 0.720, the accuracies were all greater than 0.860, etc., and the accuracies were all greater than 0.400.

Finally, we built a website to predict early PONV using the Streamlit app: (https://zhouchengmao-streamlit-app-lsvc-ad-st-app-lsvc-adab-ponv-m9ynsb.streamlit.app/). Follow these steps to predict PONV: first, upload your own data (Supplementary Fig. 1); then, select the relevant variable parameters and click on “Do Predict”; finally, obtain the predicted results (Supplementary Fig. 2). On this website, we can make predictions by inputting relevant parameters of patients individually.

## Discussions

As one of the most unpleasant experiences after surgery, PONV reduces patient satisfaction and increases hospital costs as patients remain in the hospital for extended periods of time. Machine learning can facilitate the workflow of clinicians, optimize diagnostic procedures, and reduce healthcare costs. In this study, we built a preliminary artificial intelligence algorithm model to predict early PONV. The results showed that logistic regression, SVC and adab were the three best artificial intelligence algorithms in overall performance for predicting early PONV. Additionally, haloperidol, sex, age, smoking history, and PONV history were the first 5 contributing factors in the development of early PONV.

Multiple studies have demonstrated a strong association between sex and PONV. In a study of factors influencing PONV, sex was considered an independent risk factor affecting PONV occurrence [2]. Studies have shown that different phases of the menstrual cycle lead to different incidences of PONV, possibly due to different gonadotropin and hormone levels in the body [10]. However, an extensive randomized controlled trial including 5,199 patients confirmed that there was no association between the menstrual cycle or menopausal status and PONV incidence [11]. The results of the present study supported the idea that sex is one of the main influencing factors in the occurrence of early PONV.

Several studies have also shown strong associations between haloperidol, age, smoking history and PONV history and early PONV. Additional studies have shown that age and surgery time can predict postoperative nausea and vomiting in patients undergoing oral and maxillofacial surgery [12]. Other studies have shown that being female and being young are important risk factors for postoperative nausea and vomiting in patients undergoing elective craniotomy [13]. According to some studies, PONV history, motion sickness history, and smoking history are the most influential factors for PONV [14]. The most important predictors associated with increased risk of PONV are patients being young (15 to 25 years) and non-smoking status [15]. Moreover, previous history of PONV, prolonged anesthesia and use of remifentanil during surgery have been identified as risk factors for nausea and vomiting [16]. Also, the use of haloperidol in PONV treatment has been studied extensively [17]. These findings are confirmed by our study as well.

Our study has several shortcomings: first, it is a retrospective analysis with the potential for selection bias; second, it lacks some objective variables such as biochemical tests; third, generalizability of the models remains unknown owing to the lack of multiple data sets and external validation.

## Conclusions

In summary, artificial intelligence models based on logistic regression, SVC and adab have great prospects in improving prediction accuracy and the efficiency of postoperative nausea and vomiting and early intervention. They also provide treatment decision support for high-quality postoperative rehabilitation and care.

## Supplementary Information

**Additional file 1.**

**Additional file 2: Supplementary Figure 1.** Operating interface.

**Additional file 3: Supplementary Figure 2.** The web page predicts the running results of PONV.

## Data Availability

Data are available at the BioStudies database (https://www.ebi.ac.uk/biostudies/), accession number: S-EPMC4713839.
