# Supplementary material for: Predicting early postoperative PONV using multiple machine-learning- and deep-learning-algorithms
Source: BMC Med Res Methodol. 2023 May 31;23:133. doi: 10.1186/s12874-023-01955-z (PMC10230679; doi:10.1186/s12874-023-01955-z)

In [3]:

```
f = 'pocd.csv'
pocd = pd.read_csv(f, encoding = 'gb18030')
imp = SimpleImputer(missing_values=np.NaN, strategy='mean')
imp.fit(pocd)
new_pocd = imp.transform(pocd)
new_pocd = pd.DataFrame(new_pocd)
new_pocd.columns = pocd.columns

pocd = new_pocd

pocd.describe()
```

Out[3]:

	haloperidol	sex	age	smoking	history PONV	combine Anesthesi
**count**	2617.000000	2617.000000	2617.000000	2617.000000	2617.000000	2617.00000
**mean**	0.486053	0.473443	60.891097	0.155904	0.024455	0.464654
**std**	0.499901	0.499390	13.374544	0.362833	0.154488	0.498844
**min**	0.000000	0.000000	18.000000	0.000000	0.000000	0.000000
**25%**	0.000000	0.000000	52.000000	0.000000	0.000000	0.000000
**50%**	0.000000	0.000000	63.000000	0.000000	0.000000	0.000000
**75%**	1.000000	1.000000	71.000000	0.000000	0.000000	1.000000
**max**	1.000000	1.000000	92.000000	1.000000	1.000000	1.000000

In [4]:

```
pocd.head()
```

Out[4]:

	haloperidol	sex	age	smoking	history PONV	combined Anesthesia	anesthesia Duration	sufentanil Bolus	sufent Epid
**0**	1.0	1.0	73.0	1.0	0.0	0.0	229.0	50.0	0.0
**1**	1.0	0.0	58.0	0.0	0.0	1.0	228.0	0.0	10.0
**2**	1.0	0.0	69.0	0.0	0.0	0.0	149.0	0.0	0.0
**3**	1.0	1.0	53.0	0.0	0.0	0.0	133.0	0.0	0.0
**4**	0.0	0.0	66.0	0.0	0.0	1.0	233.0	0.0	10.0

In [7]:

```
plt.figure(figsize=(22,23), dpi= 300)
sns.heatmap(pocd.corr(), xticklabels=pocd.corr().columns, yticklabels=pocd.corr().columns, cmap=
'RdBu', center=0, annot=True)

plt.title('Correlogram', fontsize=22)
plt.xticks(fontsize=12)
plt.yticks(fontsize=12)
# plt.tight_layout()
plt.savefig('Correlogram.jpg',dpi=300)
plt.show()
```

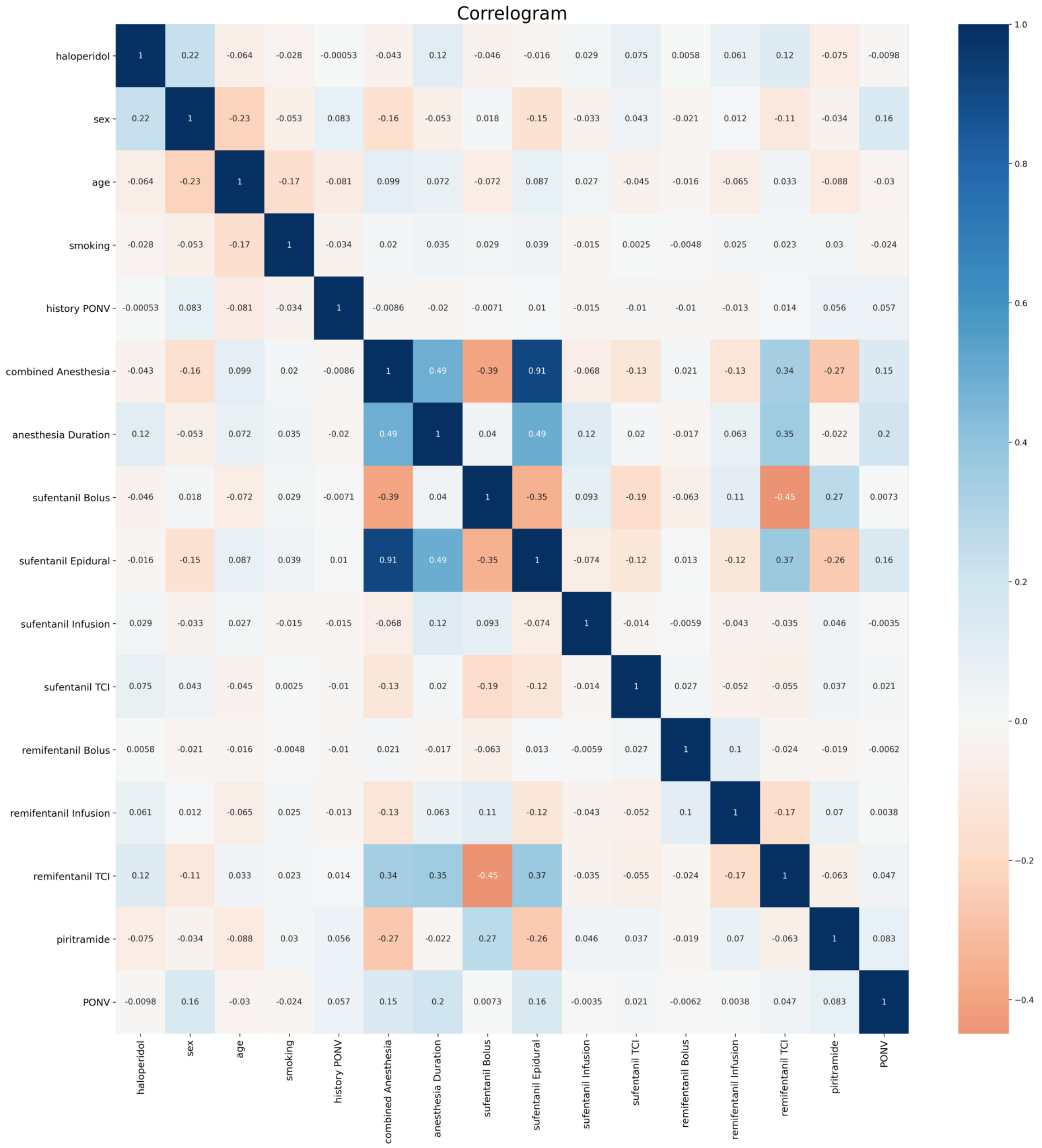

In [29]:

```
lr = LogisticRegression(penalty='l2',tol=0.0000001,C=10,fit_intercept=True,intercept_scaling=1,
max_iter=100,multi_class='ovr',verbose=0,warm_start=False,n_jobs=1)  # 逻辑回归模型
lr_result = model_fit_score(lr, X_train, y_train)
model_print(lr_result, "LogisticRegression - Train")

tr = DecisionTreeClassifier(splitter='best', max_depth=3, min_samples_split=60,min_samples_leaf
=20, min_weight_fraction_leaf=0.001,  random_state=1)  # 决策树模型
tr_result = model_fit_score(tr, X_train, y_train)
model_print(tr_result, "DecisionTreeClassifier - Train")

lsvc = LinearSVC(penalty='l2', loss='squared_hinge', dual=True, tol=0.0001, C=1.0, multi_class=
'ovr', fit_intercept=True,
                 intercept_scaling=1, class_weight=None, verbose=0, random_state=1, max_iter=10
00)
lsvc = CalibratedClassifierCV(lsvc)
lsvc_result = model_fit_score(lsvc, X_train, y_train)
model_print(lsvc_result, "LinearSVC - Train")

gnb = GaussianNB()
gnb_result = model_fit_score(gnb, X_train, y_train)
model_print(gnb_result, "gnb - Train")

knn = KNeighborsClassifier(n_neighbors=5, weights='uniform', algorithm='auto', leaf_size=30, p=2
, metric='minkowski', metric_params=None, n_jobs=1)
knn_result = model_fit_score(knn, X_train, y_train)
model_print(knn_result, "knn - Train")

adab = AdaBoostClassifier(n_estimators=50, learning_rate=1.0, algorithm='SAMME.R', random_state=
1)
adab_result = model_fit_score(adab, X_train, y_train)
model_print(adab_result, "adab - Train")
```

```
Model Performance: mean: 87.11% std: (2.36%)
[LinearSVC - Train] accuracy_score: 0.872, preci_score: 0.583, recall_score: 0.03
0, f1_score: 0.056, auc: 0.771,
 mse: 0.128, zero_one_loss_fraction: 0.128, zero_one_loss_num: 234.000, cv_score:
0.513
confusion_matrix:
 [[1590    5]
 [ 229    7]]


append_result_to_csv: ok!
Model Performance: mean: 87.11% std: (2.61%)
[MLPC - Train] accuracy_score: 0.871, preci_score: 0.000, recall_score: 0.000, f1_
score: 0.000, auc: 0.773,
 mse: 0.129, zero_one_loss_fraction: 0.129, zero_one_loss_num: 236.000, cv_score:
0.500
confusion_matrix:
 [[1595    0]
 [ 236    0]]


append_result_to_csv: ok!
Model Performance: mean: 69.57% std: (26.25%)
[gnb - Train] accuracy_score: 0.819, preci_score: 0.222, recall_score: 0.161, f1_s
core: 0.187, auc: 0.718,
 mse: 0.181, zero_one_loss_fraction: 0.181, zero_one_loss_num: 331.000, cv_score:
0.539
confusion_matrix:
 [[1462  133]
 [ 198   38]]


append_result_to_csv: ok!
Model Performance: mean: 84.76% std: (2.12%)
[knn - Train] accuracy_score: 0.886, preci_score: 0.700, recall_score: 0.208, f1_s
core: 0.320, auc: 0.888,
 mse: 0.114, zero_one_loss_fraction: 0.114, zero_one_loss_num: 208.000, cv_score:
0.597
confusion_matrix:
 [[1574   21]
 [ 187   49]]


append_result_to_csv: ok!
Model Performance: mean: 86.23% std: (2.56%)
[adab - Train] accuracy_score: 0.871, preci_score: 0.487, recall_score: 0.081, f1_
score: 0.138, auc: 0.819,
 mse: 0.129, zero_one_loss_fraction: 0.129, zero_one_loss_num: 237.000, cv_score:
0.534
confusion_matrix:
 [[1575   20]
 [ 217   19]]


append_result_to_csv: ok!
```

------------for 神经网络------------

In [46]:

```
#Create network (DNN)
def create_network_ann():
    model = Sequential()
    model.add(Dense(128, activation = 'relu', input_dim=nb_features))
    model.add(Dense(128, activation='relu'))
    model.add(Dense(1, activation="sigmoid"))
    model.summary()

    # from tensorflow.keras.optimizers import SGD
    # opt = SGD(lr=1e-3, decay=1e-5, momentum=0.3, nesterov=True)

    # model.compile(loss=loss_name, optimizer = opt, metrics=metrics)
    model.compile(loss=loss_name, optimizer="adam", metrics=metrics)
    return model

model_ann = KerasClassifier(build_fn=create_network_ann,
                                 epochs=epochs, batch_size=batch_size)
```

In [48]:

```
# clinicalInput = np.array(clinicalInput).reshape(600, 18, 1)

#Create network (CNN)
def create_network_cnn():
    model = Sequential()
    model.add(Conv1D(32, 1, input_shape=(nb_features,1)))
    model.add(Activation("relu"))
    model.add(MaxPooling1D(2))
    model.add(Conv1D(64, 1))
    model.add(Activation("relu"))
    model.add(MaxPooling1D(2))
    model.add(Conv1D(64, 1))
    model.add((Flatten()))
    model.add(Dense(1, activation="sigmoid"))
    model.summary()

    # from tensorflow.keras.optimizers import SGD
    # opt = SGD(lr=1e-3, decay=1e-5, momentum=0.3, nesterov=True)

    # model.compile(loss=loss_name, optimizer = opt, metrics=metrics)
    model.compile(loss=loss_name, optimizer="adam", metrics=metrics)
    return model

model_cnn = KerasClassifier(build_fn=create_network_cnn,
                                 epochs=epochs, batch_size=batch_size)
```

In [49]:

```
#Create network (LSTM)
def create_network_lstm():
    model = Sequential()
    model.add(LSTM(128,input_shape=(nb_features,1)))
    model.add(Dense(64))
    model.add(Dense(1, activation="sigmoid"))
    model.summary()

    # from tensorflow.keras.optimizers import SGD
    # opt = SGD(lr=1e-3, decay=1e-5, momentum=0.3, nesterov=True)

    # model.compile(loss=loss_name, optimizer = opt, metrics=metrics)
    model.compile(loss=loss_name, optimizer="adam", metrics=metrics)
    return model

model_lstm = KerasClassifier(build_fn=create_network_lstm,
                                 epochs=epochs, batch_size=batch_size)
```

In [50]:

```
# clinicalInput = np.array(clinicalInput).reshape(600, 18, 1)

#Create network (CNNRNN)
def create_network_cnnrnn():
    model = Sequential()
    model.add(Conv1D(32, 1, input_shape=(nb_features,1)))
    model.add(Activation("relu"))
    model.add(MaxPooling1D(2))
    model.add(Conv1D(64, 1))
    model.add(Activation("relu"))
    model.add(MaxPooling1D(2))
    model.add(Conv1D(64, 1))
    model.add(SimpleRNN(128, input_shape=(nb_features,1)))
    model.add(Dense(64))
    model.add((Flatten()))
    model.add(Dense(1, activation="sigmoid"))
    model.summary()

    # from tensorflow.keras.optimizers import SGD
    # opt = SGD(lr=1e-3, decay=1e-5, momentum=0.3, nesterov=True)

    # model.compile(loss=loss_name, optimizer = opt, metrics=metrics)
    model.compile(loss=loss_name, optimizer="adam", metrics=metrics)
    return model

model_cnnrnn = KerasClassifier(build_fn=create_network_cnnrnn,
                                 epochs=epochs, batch_size=batch_size)
```

------------机器学习 神经网络 合并展示------------

In [78]:

```
plt_roc_auc([
    # --单模型--
    (lr_result, 'LogisticRegression', ),
    (tr_result, 'DecisionTree', ),
    (lsvc_result, 'LinearSVC', ),
    (gnb_result, 'GaussianNB', ),
    (knn_result, 'KNeighbors', ),
    (adab_result, 'AdaBoost', ),
    (ann_result, 'DNN', ),
    (rnn_result, 'RNN', ),
    (lstm_result, 'LSTM', ),
    (cnnrnn_result, 'CNN+RNN', ),

], 'Train ROC - ALL')
```

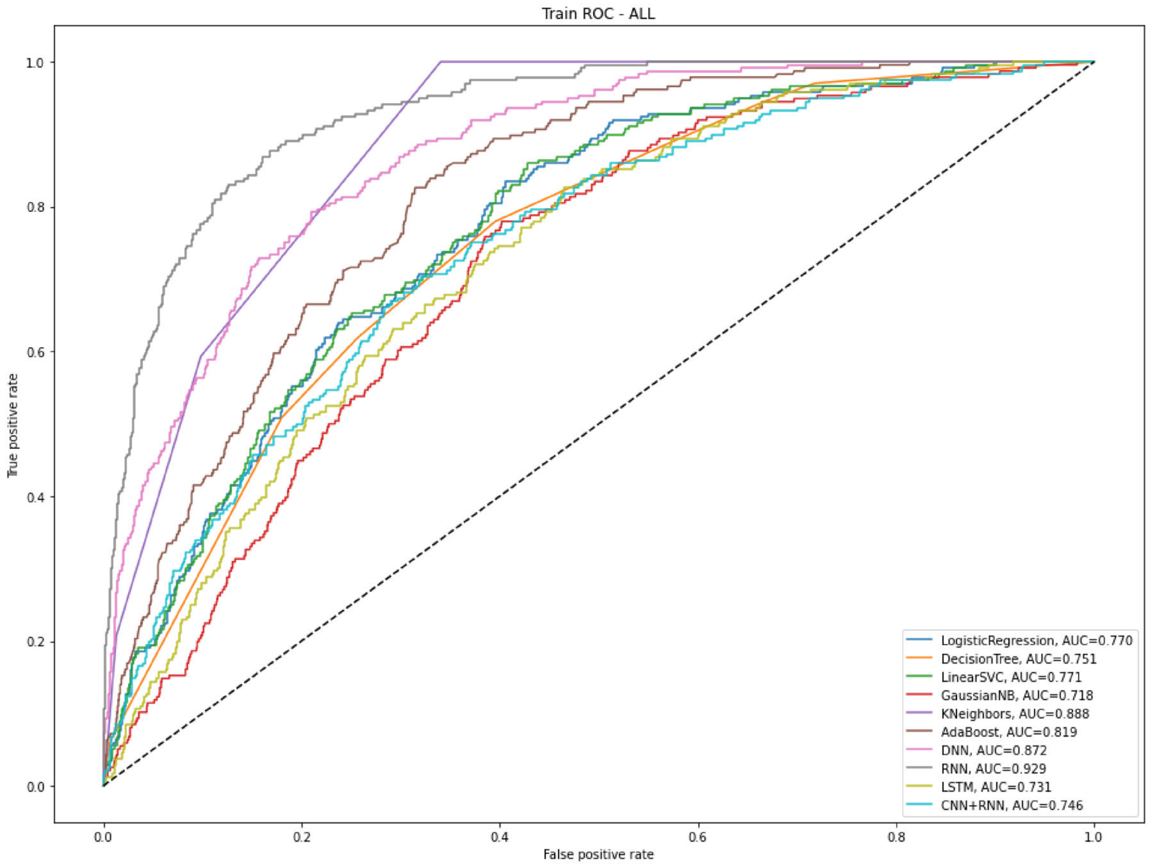

In [79]:

```
plt_roc_auc([
    (lr_test_result, 'LogisticRegression', ),
    (tr_test_result, 'DecisionTree', ),
    (lsvc_test_result, 'LinearSVC', ),
    (gnb_test_result, 'GaussianNB', ),
    (knn_test_result, 'KNeighbors', ),
    (adab_test_result, 'AdaBoost', ),
    (ann_test_result, 'DNN', ),
    (rnn_test_result, 'RNN', ),
    (lstm_test_result, 'LSTM', ),
    (cnnrnn_test_result, 'CNN+RNN', ),

], 'Test ROC - ALL')
```

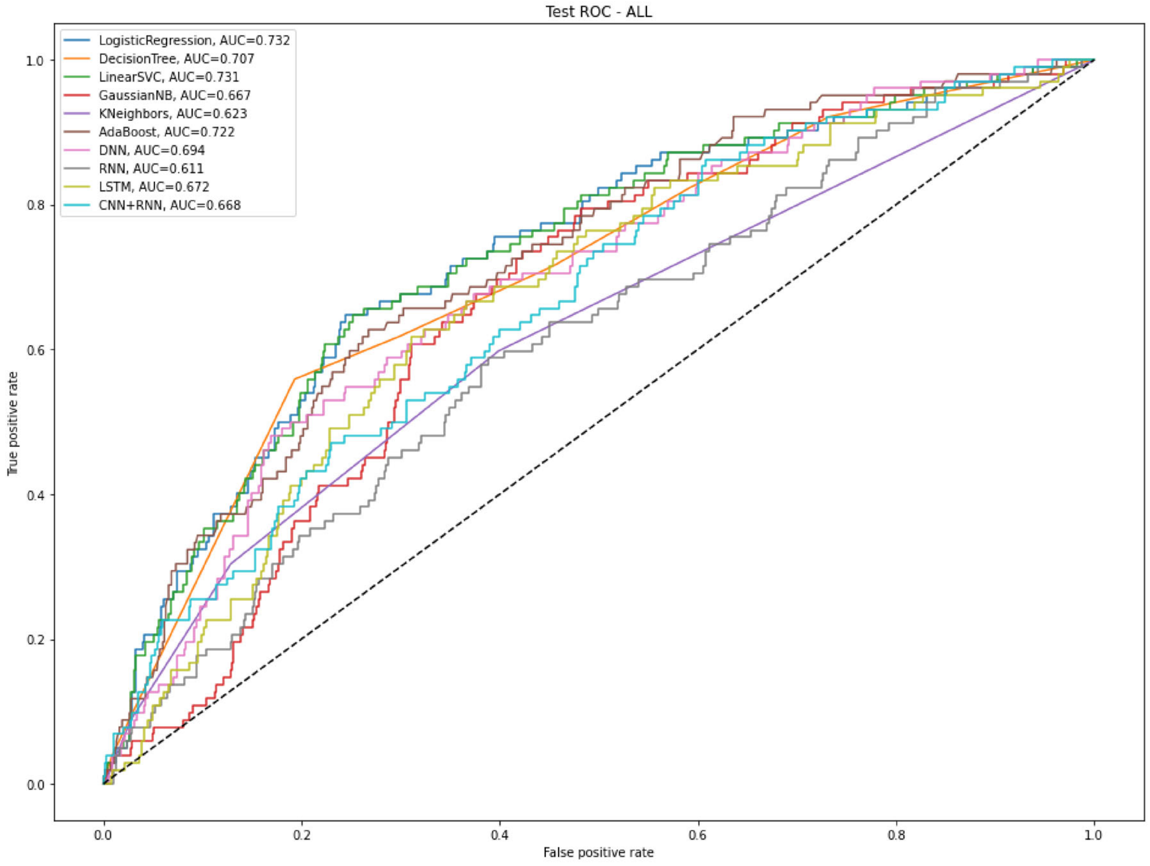

Supplement: Supplementary file 1 — Additional file 1. [file 12874_2023_1955_MOESM1_ESM.pdf]
