# Supplementary figures and images for: Predicting early postoperative PONV using multiple machine-learning- and deep-learning-algorithms
Source: BMC Med Res Methodol. 2023 May 31;23:133. doi: 10.1186/s12874-023-01955-z (PMC10230679; doi:10.1186/s12874-023-01955-z)

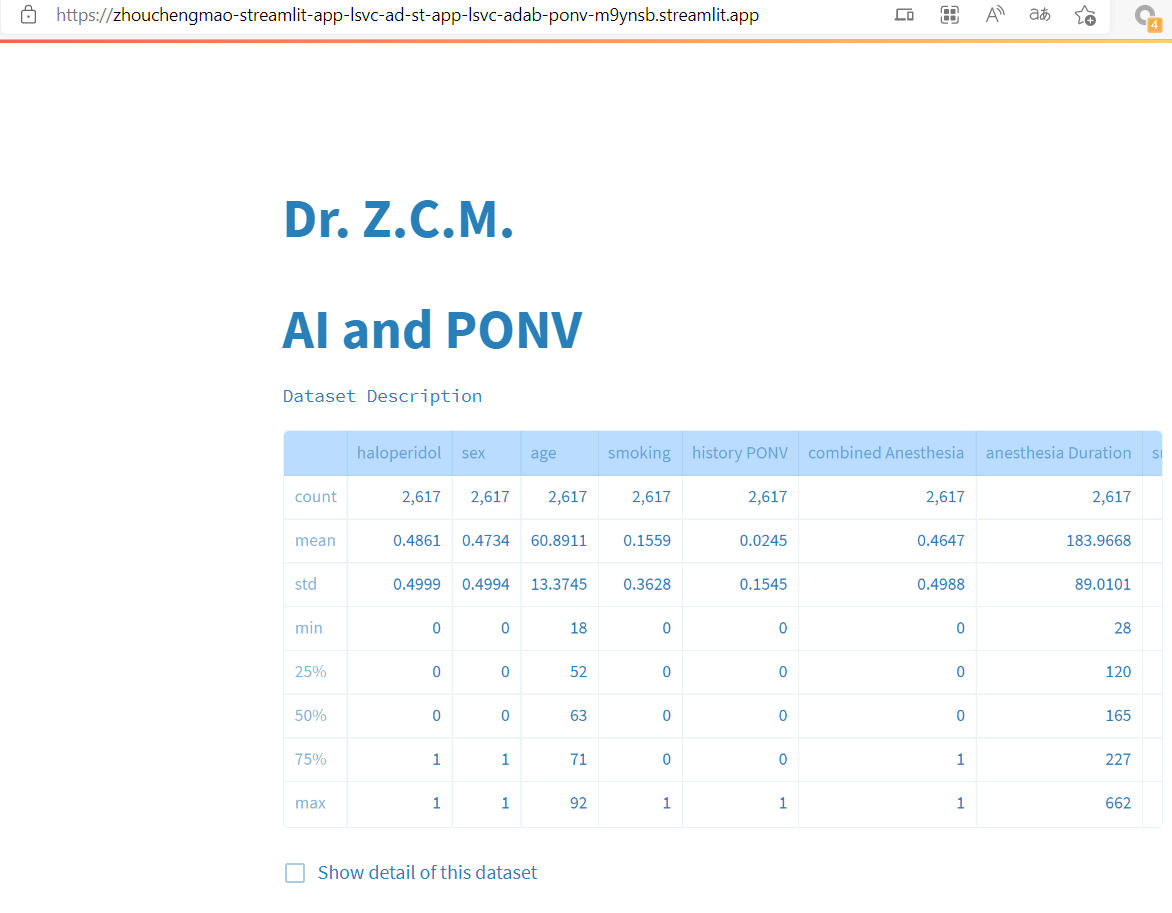


**Supplementary Figure 1 Operating interface**

Supplement: Supplementary file 2 — Additional file 2: Supplementary Figure 1. Operating interface. [file 12874_2023_1955_MOESM2_ESM.doc]

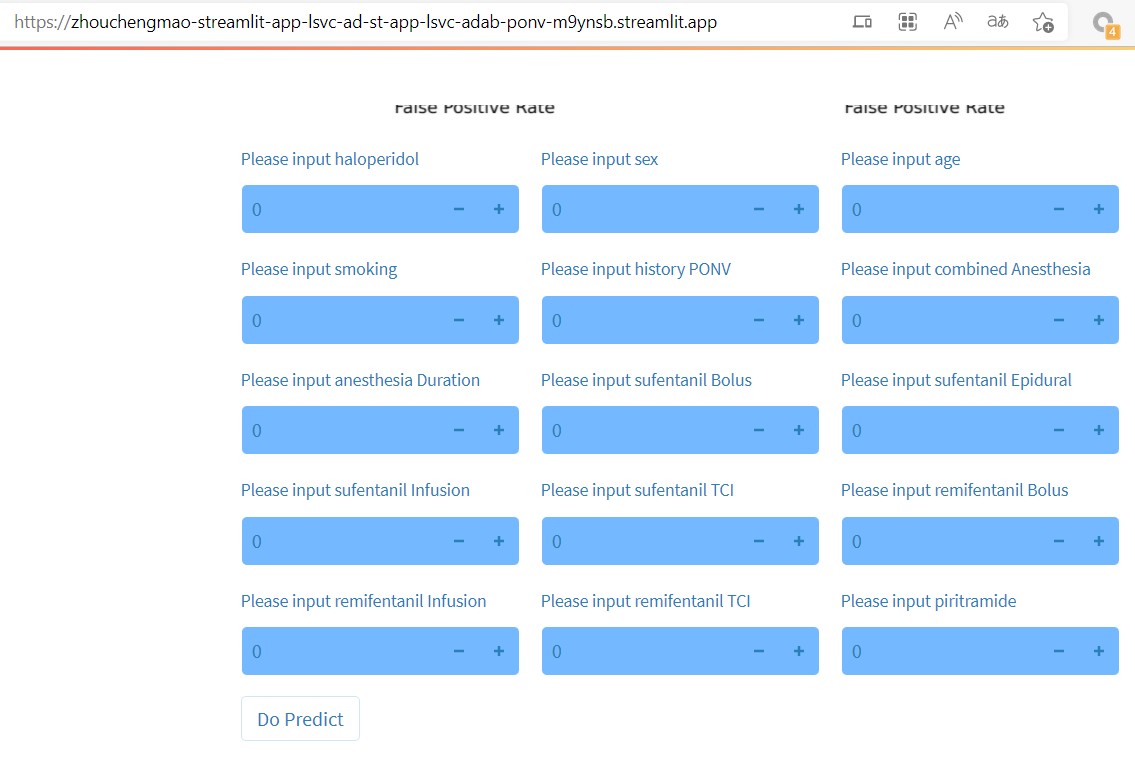


**Supplementary Figure 2 The web page predicts the running results of PONV**

Supplement: Supplementary file 3 — Additional file 3: Supplementary Figure 2. The web page predicts the running results of PONV. [file 12874_2023_1955_MOESM3_ESM.doc]
